# Pelvic abscess associated with Actinomyces species ‒ a rare post-cesarean complication

**DOI:** 10.1515/crpm-2021-0048

**Published:** 2022-03-14

**Authors:** Yuping Wang, Simone Ferrero, Shasha Li, Shisan Liu, Wah Yang

**Affiliations:** Department of Pharmacy, The First Affiliated Hospital of Jinan University, Guangzhou, P. R. China; IRCCS Ospedale Policlinico San Martino, University of Genova, Genova, Italy; Obstetrics and Gynecology Department, The First Affiliated Hospital of Jinan University, Guangzhou, P. R. China; Department of General Surgery, The First Affiliated Hospital of Jinan University, Guangzhou, P. R. China

**Keywords:** *Actinomyces odontolyticus*, pelvic abscess, post-cesarean complication

## Abstract

**Objectives:**

Pelvic actinomycotic abscess is uncommon and its presentation as a post-cesarean complication may be confused with hemorrhagic mass. It is still a disease that poses a significant diagnostic challenge. Management and prognosis are not well known for this type of infection.

**Case presentation:**

A 36-year-old woman was admitted to the hospital six days after the cesarean section with abdominal pain and dysuria. The second operation was diagnosed as pelvic abscess, debridement and drainage about 250 mL abscess. Bacterial culture of abscess confirmed as *Actinomyces odontolyticus* infection. Intravenous penicillin was given immediately, amoxicillin was taken orally for three months after discharge, and no recurrence was found after follow-up for ten months.

**Conclusions:**

Pelvic *A. odontolyticus* abscess may be confirmed through correct bacterial culture and cured by a short-term course of Amoxicillin. With prompt recognition and treatment, favorable outcomes of pelvic Actinomycotic abscess in the perinatal period could be achieved.

## Introduction

*Actinomyces odontolyticus* (*A. odontolyticus*) is a member of the family *Actinomycetaceae* and, along with *Actinomyces isruelii*, *Actinomyces nueslundii*, *Actinomyces viscosus*, and *Actinomyces meymi*, constitutes the genus *Actinomyces* [1]*.* It is a Gram-positive, microaerophilic or obligately anaerobic, rod-shaped bacterium that is part of the indigenous flora of the human oral cavity and the female genital tract [2, 3]. While *A. odontolyticus* is infrequently recognized, it can cause a diverse array of infections, including skin infections, intrauterine contraceptive device infections, bacteremia, and many others [4], [5], [6]. The most common demographic of patients affected is immunocompromised in these reports. Furthermore, to our knowledge, there are no reports of the occurrence of *A. odontolyticus* infection in non-compromised hosts, particularly during the perinatal period, even more rarely in post-cesarean patients.

Here, we report a case of a pelvic abscess caused by *A. odontolyticus* infection, which developed in the hematoma originally derived from a cesarean section.

## Case presentation

A 32-year-old woman, gravida 1, para 0, complained of continuous vaginal fluid flow for more than 2 h and bloody amniotic fluid for 20 min in the 36th week of pregnancy. After admission to our hospital, placental abruption was immediately suspected, which led to an emergency cesarean section at 36-week gestation. During the operation, one-fifth of the exfoliated surface of the placenta was found. Birthweight was 2,500 g. Apgar scores were 5 at 1 min and 10 at 5 min. The patient reported a history of hereditary glucose-6-phosphate dehydrogenase deficiency (G6PD). There was no abnormality in her medical history and allergy history. Serology of maternal blood for human immunodeficiency virus, Chlamydia, and syphilis, Group B streptococcal status was negative. She had no history of a poor dentition or intrauterine device (IUD) use. Down’s screening at 17 weeks of pregnancy found a high risk of trisomy 18 syndrome. Further amniocentesis chromosome examination showed no obvious abnormality in the 46 chromosome karyotype. Physical examination was normal, and subsequent antenatal visits were unremarkable. There was no prior history of severe illness or surgery.

**Figure 1:**
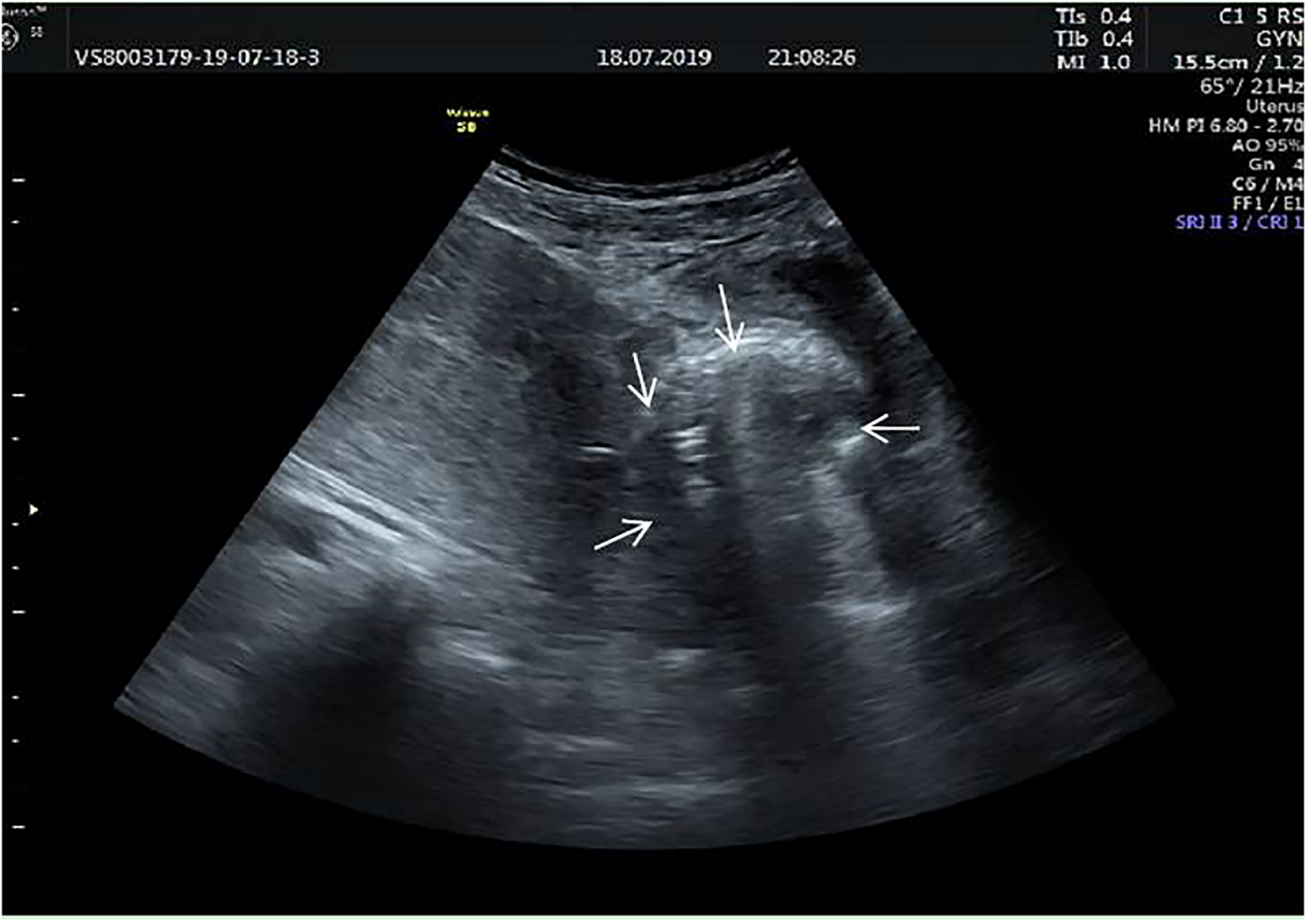
A range of about 68 × 58 mm irregular liquid dark areas was observed in the pelvic cavity on US imaging, which is located in the front of the bladder and under the scar of the lower uterine segment wall.

The patient began to experience lower abdominal pain on the second day after surgery, which was temporarily tolerable and was intermittent. Routine histologic examination of the placenta and cord was negative (Figure 1). Her postoperative hospital course was relatively uncomplicated and she was discharged home on postoperative day 3.

**Figure 2:**
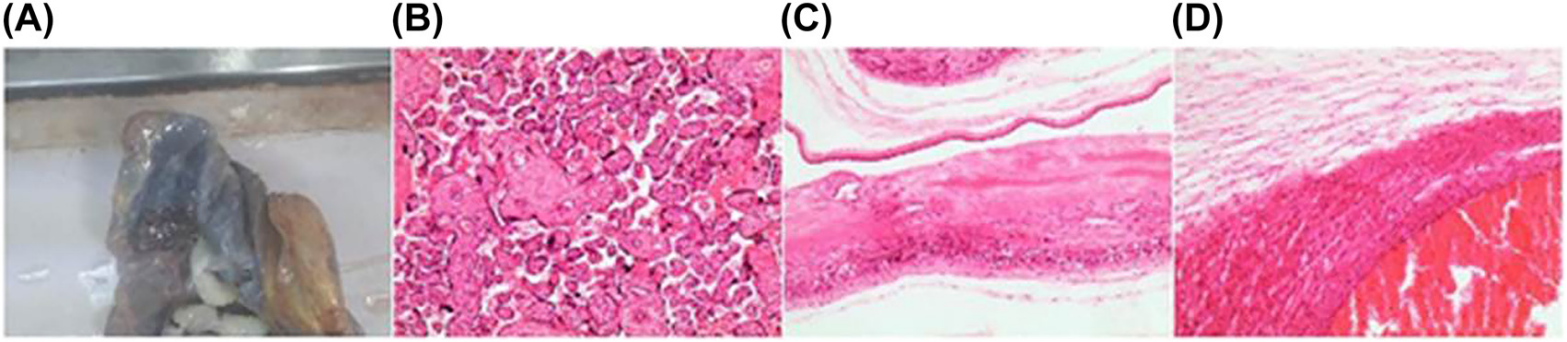
The pathology results of the placenta. (A) The size of the placenta is about 18.5 × 15 × 2 cm, multi-section dark red; (B) The villi were mature, syncytial cells proliferated, and interstitial fibrin-like material was deposited; (C) A small amount of lymphocyte infiltrates the fetal membrane; (D) Umbilical cord cross-section.

On postoperative day 6, she presented to the Emergency Department (ED) for chill but no fever, worsening lower abdominal pain, and new onset of pollakiuria, urgent urination, odynuria, and dysuria. Physical examination revealed a mass and severe tenderness in the abdominal bladder area. Gynecological examination found that the uterine cervix was smooth with no tenderness upon palpation and movement, a mass in the front wall of the vagina was palpable, and the tenderness was evident. She experienced severe pain when a catheter was inserted into the urethra. The catheter drains a small amount of urine. The patient did not have gross hematuria. A pelvic ultrasound exam revealed a mass measuring approximately 6.8 × 5.8 cm, which was located under the incision scar and in front of the bladder (Figure 2). Biological examination showed an inflammatory process with 19.820 WBC/mmy^3^ with 90.67% neutrophils and 3.93% lymphocytes, CRP 64.36 g/mm^3^. She was admitted for management of presumed pelvic hematoma.

We performed open surgery via cesarean section to remove the hematoma and check the incision site. Two cystic fluid dark areas were observed under the uterine incision during the operation. We punctured the abscess and absorbed dirty pink and foul-smelling pus. The puncture solution was reserved for aerobic and anaerobic bacterial cultures. After the uterine incision was opened layer by layer and the serosal sutures were cut off, a large amount of pus blood gushing out was observed at the lower muscle layer and the retroflection peritoneum of the bladder under the incision. Approximately 250 mL of bloody pus with a bad odor was absorbed. Then, we lavaged the space entirely, followed by the placement of a drainage tube. The patient received intravenous ceftazidime 3 g/day associated with levofloxacin 0.5 g/day for one week in the postoperative period. The puncture solution, the absorbed pus, and the drainage were respectively pumped into a blood culture bottle for aerobic and anaerobic culture. All three cultures grew the same *A. odontolyticus* species on the sixth operative day. When accurately diagnosed, the antibiotics were changed to IV penicillin 3.20 million IU every 6 h. The patient was discharged 19 days after admission on 2 g of oral Amoxicillin four times a day. The patient has been seen in the Infectious Diseases clinic on two occasions in the three months since discharge, and she remains well. Then, the patient discontinued Amoxicillin and continued to follow up for 10 months without recurrence.

## Discussion

*Actinomyces* species are a normal flora of the oral cavity, gastrointestinal and female genital tracts [7]. Post-cesarean infection can be divided into two sub-groups: surgical site infection (SSI) and endometritis [8]. Pelvic actinomycosis as the sequelae of cesarean section is rarely reported. In the gynecological literature, most female genital actinomycosis has been associated with the use of an intrauterine contraceptive device [9], some had a history of recurrent dental problems [10]. One case suspected that other intrauterine foreign bodies such as a cerclage might also be associated with invasive Actinomyces disease, because in this case, the MacDonald cerclage was placed in the mother at 19 weeks estimated gestational age [11]. In our case, the patient was free of dental disease, had an uneventful medical history and no history of IUD use. It is unlikely that the actinomycotic infection played a role in her placental abruption as the placenta and membranes showed no evidence of infection. Risk factors for pelvic actinomycosis include recent abdominal surgery, trauma, tumorigenesis when bacterium penetrates the damaged mucosa and initiates an inflammatory response leading to the formation of abscess [12]. Actinomycetes may migrate to the submucosal layer when tissue injury has occurred. Her most decisive risk factor for pelvic actinomycosis was her previous premature rupture of membranes and placental abruption based on her symptoms of abdominal pain the day after the operation, which may have been overlooked because it was similar to the pain after a cesarean section.

The initial diagnosis of an actinomycotic abscess is complex and presentation as pelvic mass is often confused with other complications after cesarean delivery [13]. This type of abscess is usually unsuspected, and thus, diagnosed and treated surgically, according to the intraoperative sulfur granules and postoperative pathological tissue identification [14]. No typical sulfur particles were seen in our case, but yielding port dark pink and foul-smelling pus, as has been reported [10, 15]. Microbiological identification of *Actinomyce*s to the species level is difficult in the clinical laboratory. Cultures require strict anaerobic conditions, which is not a routine practice at most institutions. Using 16S *rRNA* gene sequencing to identify clinically derived *Actinomyces* spp was accurate [16], but many primary hospital germ laboratories do not have such a technical test in China. Because it is a very devastating disease, the prevention and timely detection of these infections are of paramount importance. One report suggested that a biopsy should be performed in time for cases of suspected actinomycetes and a Papanicolaou smear was a simple and effective method for diagnosis of uterine actinomycosis [17]. Our experience has been similar, but fortunately, by recognizing the unwell patient and swiftly identifying *A. odontolyticus*, which are cultured by inoculating secretions into a blood culture flask, with appropriate therapy, the patient made a good recovery.

Usually, pelvic actinomycosis needs to be treated with surgery; very few cases proposed conservative treatment only with antibiotics [18]. There are no evidence-based guidelines for the treatment of actinomycosis. The traditional treatment is high-dose penicillin for 2–6 weeks after surgery, followed by oral penicillin or Amoxicillin for 6–12 months [19]. Good results with shorter courses of antibiotic treatment in stable patients have been reported. Atad, et al. [20] analyzed 11 cases of pelvic actinomycosis diagnosed and found that the duration of treatment was 12 months in six patients, six months in 3, and ≤3 months. They proposed that in cases of pelvic actinomycosis where the abscess can be completely removed surgically, a shorter period of antibiotic therapy can be effective. Here, the patient was treated with penicillin for two weeks, followed by 500 mg of oral Amoxicillin four times daily for a further three months. No recurrence was observed during the 10-month follow-up.

At present, as one of post-cesarean complications, abdominal and pelvic actinomycosis is rare domestically and internationally. This case is interesting because three of its features are unique. First, there are no documented cases of *A. odontolyticus* infection in patients undergoing cesarean-sections. Second, the diagnosis was made sure purely on positive pus culture from three different sites. Third, the source of the suspected infection is not clear. The patient had only a history of premature rupture of membranes and placental abruption and therefore chose cesarean delivery. We propose that anaerobic cultures should be performed on amniotic fluid and placentas from women who are seen in preterm labor, particularly those who have a history of miscarriages, pelvic infections IUD use, or vaginal discharge.

In clinical practice, Shiyin Hu et al. [17] suggested physicians need to raise awareness of actinomycosis and establish means of early detection or prevention of these infections. Additional cases are required to define further the epidemiologic characteristics, pathogenicity, and treatment of this organism in the perinatal period.
